# Effects of Aqua-Glycerol Uptake Facilitator Protein GlpF on Spore Germination of *Bacillus subtilis*

**DOI:** 10.3390/foods14050750

**Published:** 2025-02-22

**Authors:** Tianlin Cui, Zequn Zhang, Kangyi Mu, Yicong Shi, Fang Chen, Li Dong, Xiaosong Hu

**Affiliations:** 1 College of Food Science and Nutritional Engineering, National Engineering Research Center for Fruit and Vegetable Processing, Key Laboratory of Fruits and Vegetables Processing, Ministry of Agriculture, Engineering Research Centre for Fruits and Vegetables Processing, Ministry of Education, China Agricultural University, Beijing 100083, China; tianlincui@163.com (T.C.); mkys1999@163.com (K.M.); s20223061139@cau.edu.cn (Y.S.); chenfangch@sina.com (F.C.); 2 College of Food Science and Technology, Henan Agricultural University, Zhengzhou 450002, China; zqzhang1995@126.com

**Keywords:** *Bacillus subtilis*, spore germination, GlpF, high pressure

## Abstract

Killing spores is an important challenge for the development of the food industry. After germination, the resistance of spores disappears and they are more easily killed, which is currently the main strategy for their destruction. Therefore, study of the mechanism of spore germination is of great significance for improving methods of spore inactivation. Previous studies have shown that the hydration of the spore core region, accompanied by the disappearance of bacterial spore resistance, is a key step in the germination pathway of bacterial spores. However, the specific mechanism of this process has been studied very little. In this study, *Bacillus subtilis* PY79 was used as a model strain, and its single water glycerol channel protein (GlpF) was regarded as a starting point to explore the mechanism of water transport during spore germination. First, we constructed *glpF* mutants and overexpression strains and discovered that the deletion of *glpF* did not affect the growth of bacterial vegetative cells and spores. Further germination experiments on the spores of the *glpF*-deficient strain through detecting calcium dipicolinate and absorbance of spores showed that the germination rate of the mutant strain spores increased, while increasing the water activity did not affect the results caused by *glpF* deletion. Meanwhile, overexpressed *glpF* affected the permeability of the spore coat. Finally, when treating spores with ultra-high pressure, the spores lacking *glpF* were more likely to be inactivated. The above results have suggested that the *glpF* gene plays an important role in spore germination.

## 1. Introduction

Spores are formed by certain bacteria as a survival strategy in response to environmental stress, such as high temperature, cold, and pressure [1], which contribute to the multilayered structure of the spore. Some spores are always difficult to eliminate during food sterilization treatments [2], including desiccation, freezing, thawing, heat, radiation, and using various chemicals [3,4]. Viable spores can germinate and grow in foods under suitable conditions, which can damage the nutritional components and cause food spoilage, even posing a risk to human health [5,6]. According to statistics, the *Bacillus cereus* toxin is estimated to be responsible for 16–20% of food poisoning outbreaks worldwide, caused by spore-forming *glpf* species [7,8]. Bacteria poisoning typically occurs when people eat foods that have not undergone hygienic processing [9]. Due to their extreme resistance to treatment [2], spores pose a significant threat during food processing and storage. Therefore, it is crucial to improve and advance food sterilization technologies to study the mechanisms of spore sporulation and germination.

The resistance and dormancy of spores can be attributed to several unique characteristics, in which a low water content within the core is a notable feature. After being produced within the mother cell compartment of a sporulating cell, spores remain metabolically dormant and partially dehydrated, with a water content as low as 25% by weight [10]. The maintenance of this low core water content is attributed to two reasons. First, developing spores incorporate the small molecule dipicolinic acid (DPA) into their core during sporulation [11]. Previous studies have indicated that the DPA-less spore core region appears to be more hydrated than that of spores with DPA, which suggests that DPA could help to maintain the low core water content [12]. Second, the spore cortex, made of peptidoglycan, acts like a strong and rigid cage [13]. It exerts inward pressure on the spore, counteracting the osmotic pressure from the external environment, thus helping to maintain low water activity [10].

However, the germination of spores can lead to the transition from low to high water activity, resulting in the loss of resistance. When germinating agents like L-alanine, L-valine, and L-asparagine-glucose-fructose-potassium (AGFK) are present, spores rapidly initiate the germination process [14]. With the activation of germination factors, such as GerA and GerB, DPA is released from the spore core, which is a critical event, leading to partial core hydration. Subsequently, cortex peptidoglycan hydrolysis causes an approximately two-fold expansion of the spore core due to further water uptake [15]. During spore germination, the spore core transitions from an extremely low water content to being completely hydrated. The moisture content varies across different parts of the spore, with the core being less hydrated than the cortex [16]. This difference in hydration levels is inversely related to the spore’s heat resistance [17,18]. The inner membrane surrounding the spore core serves as the primary barrier of water exchange [19,20,21] and the removal or damage of the spore coat can increase the rate of water exchange in the core region [22]. Additionally, the biofilm of *Bacillus subtilis* contains highly permeable transport channels that facilitate liquid penetration [23]. These findings have suggested the presence of specialized pathways through which water could be transported in the outer membrane of the spore core, which could play a crucial role in spore germination. However, the functional mechanism by which water enters the spore during germination remains unclear.

Multiple studies have found that microorganisms possess two types of aquaporin proteins: aquaporins (AQPs) and the water-glycerol transporter protein GlpF, both belonging to the major intrinsic protein (MIP) family [24]. GlpF is a member of the aquaporin family, facilitating the selective transport of small molecules, such as water and glycerol, across the cell membrane, driven by the concentration gradient of the substrate [25]. According to the prevailing viewpoint, aquaporins function as continuously open pores in their “open state,” allowing water molecules to permeate in a single-file manner [26]. It has been reported that aquaporin-Z (AqpZ), an orthodox *Escherichia coli* water channel, exhibits greater osmotic permeability than GlpF [27,28,29,30]. The narrow pores of AQPs exhibit remarkable permeability, conducting water in a single-file manner near the diffusion limit of bulk water, while maintaining perfect selectivity [27]. Interestingly, only one water channel protein, GlpF, is found in the genomes of many spore-producing bacteria. This suggests that *glpF* may be an essential gene involved in the water transport function during spore germination.

Therefore, this study used the model organism *B. subtilis* PY79 as the research object for investigating the effect of *glpF* in spore germination. We constructed genetically engineered strains related to *glpF* and analyzed the spore germination under different germinants and external conditions. Additionally, the survival rate of the spores after high-pressure treatment was tested.

## 2. Materials and Methods

### 2.1. Strains Construction

All strains were derived from *B. subtilis* PY79 [31]. The strains and plasmids are listed, along with strategies used for their construction (Table 1). The *E. coli* strain used for plasmid constructions was DH5α. Cells were grown in Luria–Bertani (LB) medium. The antibiotics and additional substances used were ampicillin (100 µg·ml^−1^); kanamycin (5 µg·ml^−1^); spectinomycin (60 µg·ml^−1^); and IPTG (100 mM).

The deletion of the *glpF* gene in the PY79 strain was achieved using a long-flanking homology region PCR method based on the principle of double-crossover homologous recombination. Competent cells of the PY79 strain were transformed with the DNA fragment consisting of the upstream and downstream homologous arms fused with the resistance gene. Then, strain BS01 was successfully constructed.

The pDR111-*glpF* plasmid, containing the *glpF* gene fused to the IPTG-dependent *Phyper-spank* promoter flanked by *amyE* front and back regions, was constructed by PCR amplifying the *glpF* open reading frame and NheI/HindIII restriction sites. The constructions were validated by DNA sequencing. Competent cells were prepared using MC buffer [32] (14.036 g·L^−1^ K_2_HPO_4_, 5.239 g·L^−1^ KH_2_PO_4_, 20 g·L^−1^ glucose, 3 mmol·L^−1^ trisoduim citrate, 22 mg·L^−1^ ferric ammonium citrate, 1 g·L^−1^ casein hydrolysate, 2 g·L^−1^ potassium glutamate). The plasmid pBS1 was transformed into competent cells, and after validation, strain BS02 was obtained.

### 2.2. Sporulation and Purification of Spore

Sporulation was induced at 37 °C by suspending cells in Schaeffer’s liquid medium (Difco Sporulation Medium, Maastricht, The Netherlands). Spore purification was performed as previously described [33] with modifications. To generate spores for germination assays, cells were grown in liquid LB medium at 37 °C for night, and then spread on liquid DSM and incubated for 30 h at 37 °C. Spores from each tube were centrifuged to collect, washed 6 times with ddH_2_O, and then resuspended. The suspension was layered on top of 1 mL 50% histodenz (Sigma-Aldrich, St. Louis, MO, USA) in a microfuge tube and the step gradient was centrifuged at 12,000× *g* for 10 min at room temperature. The pellet containing mature, phase-bright spores was collected and washed 6 times with ddH_2_O. The spores consisted of 99% dormant phase-bright spores as determined by phase-contrast microscopy and were stored at −80 °C for later use.

### 2.3. Spore Germination and Reduction in Optical Density (OD_600_) Assay

Spore germination was induced by L-Alanine (10 mM), L-Valine (10 mM) or AGFK (10 mM L-Asparagine, 10 mM D-glucose, 10 mM D-fructose, and 10 mM KCl) at 37 °C. Before determination, histodenz-purified phase-bright spores were heat-activated at 65 °C for 30 min [34], followed by cooling at room temperature for 5 min. A volume of 100 μL of spore suspension was then transferred to a clear, flat-bottom 96-well plate. An equal volume of nutrients or buffer as described above was added to the spore suspension for a final OD_600_ of 0.5. The OD_600_ was monitored every 5 min using a multi-mode plate reader (SpectraMax iD5, Molecular Devices, San Jose, CA, USA). The plate was maintained at 37 °C between measurements. Each experiment was repeated at least 3 times, and a representative curve is shown.

### 2.4. Spore Germination and DPA Measurements

DPA release was measured as described [35] previously with modifications. Spores at OD_600_ of 0.5 were germinated at 37 °C with germinates in a 96-well plate in 200 μL of 25 mM HEPES (pH 7.4) supplemented with 50 mM TbCl_3_. DPA release was monitored by quantifying Tb^3+^-DPA fluorescence at 545 nm with excitation at 270 nm every 5 min for 2 h in a multi-mode plate reader. All samples and conditions were tested in technical triplicate.

### 2.5. Microscopy

Light microscopy was carried out as described previously [36]. The germination of large numbers of individual spores can also be analyzed simultaneously using phase-contrast microscopy. Briefly, bacterial cells (0.5 mL) were collected by centrifugation and resuspended in 50 μL of ddH_2_O. For time-lapse experiments, a drop of spore suspension was placed on the surface of 1% agarose pads supplemented with 10 mM L-alanine and air-dried so that the spores adhered firmly on the microscope slide. Then, the spores were incubated in a chamber where temperature was maintained at 37 °C with Pecon cage incubator for temperature control (Zeiss). The full-view field of the phase-contrast images of large numbers of spores germinating on the cover slips was recorded at a rate of 1 frame per 60 s. Samples were photographed using Axio Observer Z1 (Zeiss) and using the Axiocam 503 color camera and accompanying image processing software ZEISS ZEN 2.6 (blue vision). Image analysis and processing were conducted using ImageJ 1.53p (National Institutes of Health, Bethesda, MD, USA).

### 2.6. High-Pressure Treatment

Spores (OD_600_ of 0.5) were suspended in 1 mL of ddH_2_O and sealed in sterile flexible plastic bags. Spore suspensions were treated at 200 MPa at 80 °C for 15 min in a high-pressure-assisted thermal device (Bao Tou KeFa High Pressure Technology Co., Ltd., Baotou, China) with a 5 L vessel and water as the pressure-transmitting fluid. Subsequently, the quantity of surviving spores was counted using the gradient dilution plate method, which involved serially diluting the spores with ddH_2_O [37]. All plates were incubated at 37 °C for 12–18 h. In order to obtain the initial spore counts for the experiment, untreated spores were employed as the control condition. Following treatment, the spore survival log amount [log10 (N_0_/N_t_)] was calculated, where N_0_ represents the starting spore count and N_t_ represents the spore count following treatment. All experiments presented in the text figures were from one of three biological replicates.

### 2.7. Phylogenetic Analysis

We performed a detailed phylogenetic analysis on the amino acid sequences extracted from UniProt (https://www.uniprot.org). The dataset includes 7 water-transporting aquaporins (AqpZ) and 203 glycerol-transporting aquaglyceroporins (GlpF) from representative spore-forming species. ClustalW implemented in MEGAX was used for multiple sequence alignment and the phylogenetic tree was constructed using the maximum likelihood (ML) method with bootstrap values of 1000 replicates.

### 2.8. Statistical Analysis

All analyses were performed using GraphPad Prism 8 (Version 8.0.2). Statistical significance was assessed using the original false discovery rate (FDR) control method proposed by Benjamini and Hochberg. The significance level was *p* < 0.05. Data in the figures are presented as the mean ± standard deviation of three replicates. Figure and data fitting were performed using RStudio (Version 2022.12.0).

## 3. Results

### 3.1. Phylogenetic Analysis of GlpF in Spore-Producing Bacteria

**Figure 1:**
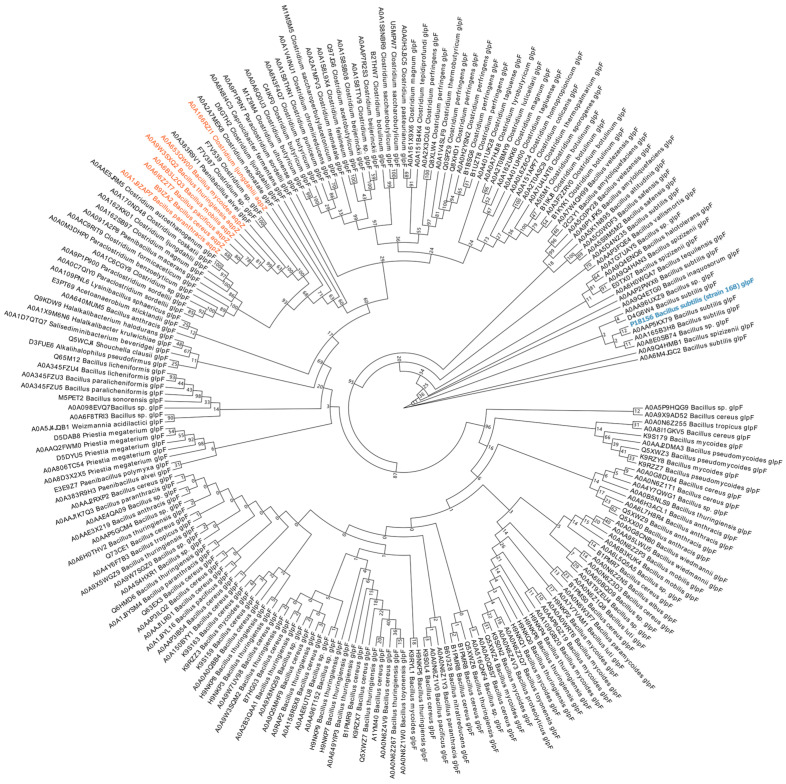
Phylogenetic analysis of aquaporins in spore-forming bacteria. Different colors represent different types of aquaporins (red, AqpZ; black, GlpF; blue, GlpF of *Bacillus subtilis*).

The inner membrane of spores acts as a permeability barrier, with a lipid composition similar to that of vegetative cells [38]. Over 900 proteins have been identified in the *B. subtilis* spore inner membrane, including those encoded by the *glp* operon [39]. We performed phylogenetic analysis of the spore-forming aquaporins, including 7 AqpZ and 203 GlpF from the representative species (Figure 1). The sequence consistency between GlpF (*B. subtilis* strain 168) and AqpZ is relatively moderate, reaching 30%. Due to only one water channel protein, GlpF, being found in the genome of *B. subtilis*, and given the structure of the spore [40], the GlpF protein was localized on the inner membrane of the spore. A previous study reported that the cell membrane channel protein GlpF could be required for glycerol uptake in vegetative cells of *B. subtilis* [41]. However, whether GlpF has the ability to transport water molecules, and its role in spore germination, has not been investigated.

### 3.2. Impact of GlpF on Spore Germination and DPA Release in B. subtilis

**Figure 2:**
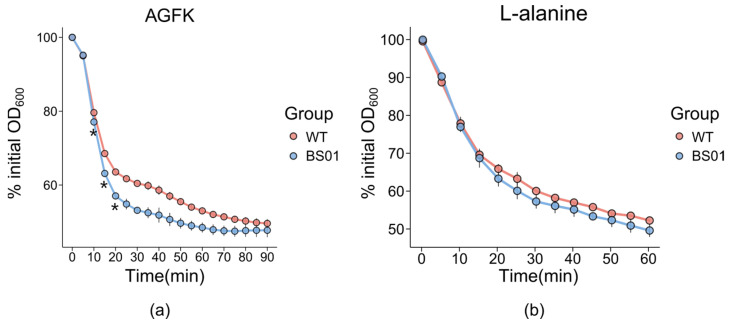
Effect of *glpF* on spore germination in *B. subtilis* strains WT and BS01. Spore germination was monitored by the change in optical density with germinants AGFK (**a**,**b**) L-alanine. Values shown are the mean values and standard deviations of triplicate measurements in three experiments (*, *p* < 0.05).

To determine whether the function of the *glpF* is crucial for germination in *B. subtilis*, we deleted the *glpF* gene in *B. subtilis* PY79 strain (wild type, WT) and obtained the mutant strain Δ*glpF* (strain BS01). The absence of GlpF protein does not affect the growth of the vegetative cells. To study the effect of the *glpF* gene on the germination of *B. subtilis* spores, we added germinants to the spore suspension and measured the decrease in absorbance using a spectrophotometer to characterize spore germination. Interestingly, the germination of BS01 strain spores commenced earlier than that of the wild-type spores, beginning 10 min after the addition of the germinant AGFK (Figure 2a). Only a slight acceleration was observed in the BS01 strain compared to the wild-type strain with the germinant L-alanine (Figure 2b). These results indicated that the absence of *glpF* could accelerate the germination process.

**Figure 3:**
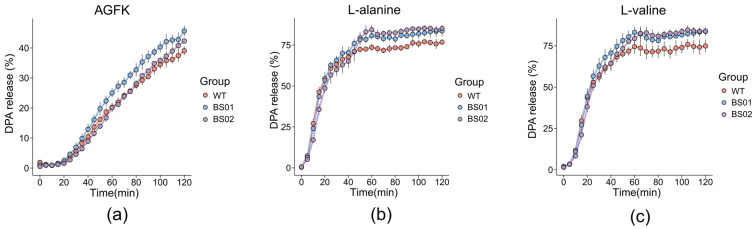
Effect of *glpF* on DPA release during spore germination. Spores of PY79 (WT), BS01 (Δ*glpF*), and BS02 (*glpF* overexpression) strains were incubated with germinants to trigger germination. DPA release to the medium was determined by Tb-DPA assay. Presented are relative fluorescence units (RFUs) measured at 545 nm with excitation at 270 nm. Shown is a representative experiment out of three independent biological repeats. The germinants used were (**a**) AGFK, (**b**) L-alanine, and (**c**) L-valine.

In addition, the release of DPA is one of the key events during the early stages of spore germination. The prevailing view suggests that DPA release is closely correlated with subsequent core hydration [42]. Consistent with previous findings, the BS01 strain of *B. subtilis* exhibited a faster DPA release rate compared to the wild type when exposed to AGFK (Figure 3a). We also observed a slight acceleration in germination with *glpF* deletion, along with the germinants L-alanine and L-valine (Figure 3b,c). Considering that inhibiting water transport during germination should make it more difficult for spores to germinate, we speculate that *glpF* might have contributed to spore formation by causing flaws in the resulting spores, which would hasten germination. So, a GlpF overexpression strain (BS02) was constructed, which induced GlpF overexpression during sporulation by adding IPTG. Through detecting the release of DPA, it was observed that their germination would slow down in the presence of various germinators including AGFK, L-val, and L-ala.

**Figure 4:**
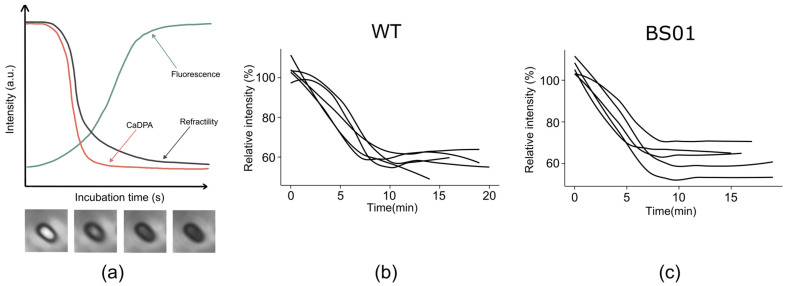
Germination plots of a single *B. subtilis* PY79 spore under a phase-contrast microscope. (**a**) The schematic diagram of CaDPA, refractility, and fluorescence intensities as a function of germination time. (**b**) PY79 (WT) and (**c**) BS01 (Δ*glpF*) are single spores under a differential interference contrast (DIC) microscope, respectively. The CaDPA level is calculated from the intensity of the Raman. Refractility refers to the intensity value under a DIC microscope. Fluorescence refers to the intensity value of the CaDPA and TbCl_3_ compound fluorescence.

Previous studies have shown that the real-time release of CaDPA is strongly correlated with the transition of spores from phase-bright to phase-dark during germination [43]. To determine whether *glpF* affects the DPA release process, we characterized the dynamics of DPA release by observing and quantifying the brightness changes during the phase transition of spore germination under a microscope. Our results indicated that the absence of *glpF* does not affect the process of spore darkening after DPA release has begun in individual spores (Figure 4). Therefore, these results imply that the loss of GlpF affects the germination of spores, mainly accelerating the progress of germination.

### 3.3. Effects of GlpF on DPA Release in Different Genotypes of B. subtilis with Osmotic Stress

**Figure 5:**
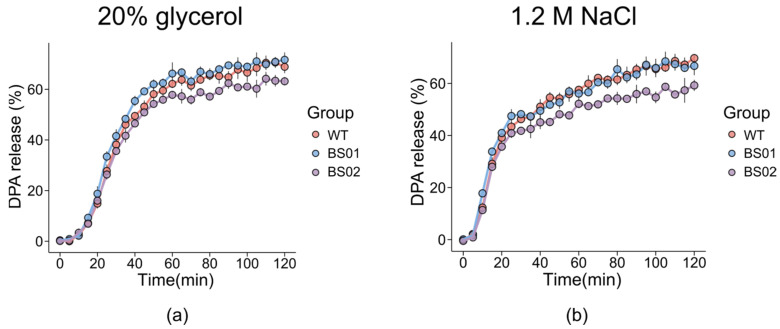
Effects of GlpF protein on DPA release during spore germination under hypo-osmotic conditions of (**a**) 20% glycerol and (**b**) 1.2M NaCl. Spores of PY79 (WT), BS01 (Δ*glpF*), and BS02 (*glpF* overexpression) strains were incubated with L-alanine to trigger germination.

GlpF, the glycerol facilitator in bacteria, is responsible for the uptake of water, glycerol, and other small alditols into the cell [44]. Previous studies have indicated that GlpF, as a water/glycerol transporter, is essential for the survival of fungi under conditions of high osmotic stress [45]. And reducing the water activity of germination incubations can inhibit bacterial spore germination, with the extent of suppression dependent on the solute used to decrease water activity (aw) [46]. Therefore, NaCl and glycerol were selected as the osmotic media, and the spore germination at a high concentration was analyzed. The results revealed that in both the 20% glycerol and 1.2M NaCl solutions, the DPA release from the overexpressing strain was lower than that of the wild type (Figure 5). Specifically, for the BS01 strain, its DPA release rate in the 20% glycerol solution was significantly higher than that of the wild type from 0 to 120 min after the treatment of L-alanine. In the 1.2 M NaCl solution, its DPA release rate exceeded that of the wild type only during the first 40 min of germination.

### 3.4. Impact of GlpF on Spore Resistance and Membrane Structure

**Figure 6:**
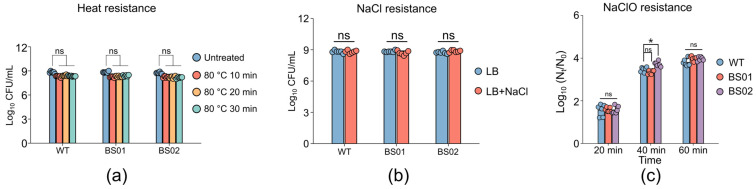
Resistance properties of spores. Moist heat (80 °C) resistance (**a**), NaClO (0.15%) resistance (**b**), and NaCl (6%) resistance (**c**) of PY79 (WT), BS01 (Δ*glpF*), and BS02 (*glpF* overexpression) strain spores (*, *p* < 0.05; ns, not significant).

To explore the reasons for the impact of GlpF on spore germination, the resistance and membrane structure of spores with different genotypes were analyzed. Initially, we examined the effects of 10, 20, and 30 min of treatment at moderate temperature (80 °C) on the inactivation of spores of *B. subtilis* strains WT, BS01, and BS02. The plate counting experiment results indicated that there were no significant differences in the heat resistance of the spores of the BS01 and BS02 strains compared to those of the wild type (Figure 6a). Previous studies have suggested that the heat resistance of spores can reflect the integrity of the cortex [42]. Our results indicated that heat treatment has no effect on spore resistance, indicating that the deletion or complementation of GlpF does not affect the spore cortex. In addition, we inoculated the three types of spores on LB plates supplemented with 6% (*w*/*v*) NaCl and observed normal growth in all the strains (Figure 6b), indicating that their inner membranes remained unaffected. However, after treating the three types of spores with 0.15% NaClO for 20, 40, and 60 min, the BS02 strain exhibited greater sensitivity to NaClO compared to the wild-type spores, particularly after 40 min of treatment (Figure 6c). This indicated that the spore coat of the BS02 strain should be destroyed. Overall, these results suggested that GlpF may play a key role in the structure and function of spores.

### 3.5. Effect of Pressure–Temperature Treatment on Spore Inactivation

**Figure 7:**
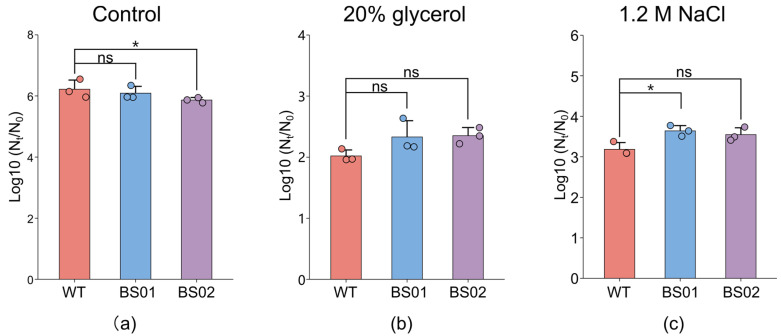
Effect of *glpF* on spore inactivation with the combined treatment of temperature and pressure in hypo-osmotic conditions of (**a**) water, (**b**) 20% glycerol, and (**c**) 1.2 M NaCl. Spores of PY79 (WT), BS01 (Δ*glpF*), and BS02 (*glpF* overexpression) strains were treated at 80 °C and 200 MPa for 15 min to induce inactivation (*, *p* < 0.05; ns, not significant).

Our preliminary results found that the treatment of 200 MPa and 80 °C for 15 min effectively sterilized the spores [47]. To examine the inactivation effect of the combined treatment of pressure and temperature (PT) on three types of spores under different osmotic pressures, plate counting experiments were conducted after high-pressure treatment in water, 20% glycerol, and 1.2 M NaCl. The results (Figure 7) showed that, in the aqueous solution, the numbers of killed spores for the three kinds of spores were 6.22, 6.09, and 5.86 log, respectively. The numbers of killed spores in the 20% glycerol solution were 2.02, 2.22, and 2.35 log, and the PT treatment in 1.2 M NaCl for 15 min led to spore reductions of 3.18, 3.64, and 3.55 log for the WT, BS01, and BS02 strains, respectively. Among them, the number of spores killed in the aqueous solution was the highest, while that in the glycerol solution was the least. In addition, there was no significant difference in the number of inactivated spores among the three kinds of spores in the 20% glycerol solution, but there were differences in the spores in the aqueous and 1.2 M NaCl solutions. In the aqueous solution, compared with the wild type, the spores of the overexpressing strain were less likely to be killed. However, the BS01 strain should be more easily inactivated with the PT treatment in 1.2 M NaCl for 15 min compared with the wild-type strains. These results indicated that spore inactivation with the PT treatment would be associated with the presence of the *glpF* gene. Moreover, the data suggested that spore inactivation depends on the osmolality of the solution.

## 4. Discussion

Dormant bacterial spores can remain inactive for decades but rapidly awaken, restoring metabolism and growth in response to nutrients [48]. In this regard, the water change in the spore core region is a crucial part. However, the mechanism of water transport during spore germination is still unknown. In this study, the characteristics and function of aquaporin GlpF in spore germination were detected. The absence of GlpF protein would not only accelerate spore germination with various germinants, but also lead to the disruption of the spore coat. Therefore, the aquaporin GlpF has a universal influence on spore germination.

Previous research has found a single stable conformation of AqpZ [26], which adopts a permanently open conformation. The opening and closing of the water channel are not regulated and affected by environmental conditions. But the spore germination process is highly ordered and controlled, which may account for the absence of AqpZ in the majority of the spore genomes. Additionally, the phenomenon of the accelerated spore germination observed following the deletion of GlpF is notable. GlpF is a membrane channel protein that affects the membrane structure; the mutant of *glpF* makes the spores more sensitive to external environments and more easily triggered to germinate. Both vegetative cells and spores include GlpF, which contributes to water transport during sporulation. The spore may use additional defense mechanisms to make up for the loss of *glpF*, guaranteeing that genetic information is passed on within the spore. Indirectly, this modification makes spore germination more sensitive [49]. Meanwhile, it has been observed that spores overexpressing GlpF exhibit reduced resistance to NaClO, potentially impacting the structure of the spore coat [50,51]. The *B. subtilis* spore coat is a multilayered protective structure composed of more than 70 different proteins [13]. As germinants will reach their receptors through traversing the spore coat and cortex, the permeability of the spore coat could influence germination [40]. The spore coat plays a key role in maintaining the low water permeability of the core [19]. We found that altering the abundance of GlpF protein could affect the permeability of the spore coat. Consequently, spores deficient in GlpF germinate faster, perhaps as a result of an impact on the spore coat’s permeability, which affects the germination rate. However, the effect of GlpF protein on the structural changes in spores remains to be explored. In addition, according to earlier research, GlpF would influence spore germination in two potential ways. Firstly, during sporulation, it facilitates controlled water efflux for core dehydration. This may impact the osmoregulatory balance of the forespore, causing changes in gene expression and forespore structure, and thus influencing germination [52]. Secondly, during germination, GlpF may influence gene expression and protein synthesis. Additionally, the downregulation of GlpF allows for rapid hydration through alternative channels when germination occurs [46]. DPA release is a critical step in the spore germination process. Initially, a small amount of DPA is released, followed by a decrease in the spore brightness. When DPA is fully released, the core becomes hydrated, causing the spore to darken completely. Our previous results revealed that deleting GlpF led to a delayed start of DPA release (Figure 3). Nevertheless, the microscopic observations indicated that there were no significant differences between the WT and BS01 stains in the process of spore darkening, which occurs subsequent to DPA release (Figure 4), suggesting that the absence of *glpF* has little impact once DPA is released. This indicates that GlpF influences the timing of germination initiation within a spore population but does not impact the molecular dynamics of DPA release during the germination process. This involves complex mechanisms in triggering germination and may be related to other factors including nutrient perception and germination commitment [35].

Moderate high-pressure treatment can induce spore germination [53]. The spores of the *glpF* mutant are more easily inactivated, which may be related to their increased propensity for germination. GR (GerA, GerB, and GerK) family receptors embedded in the spore membrane are required for sensing amino acids, sugars, and/or nucleosides [54]. They are an essential protein family required for triggering the germination program. Previous studies have shown that moderate high pressure can induce spore germination, and that spores germinated with HPP exhibit changes similar to those during nutrient germination, including Ca^2+^-DPA release [53]. The rates of pressure germination of spores lacking nutrient receptors were extremely low [55]. These results suggest that HPP induces spore germination through GRs. Consistent with our findings, the spores lacking *glpF* germinated more quickly than the wild-type spores, and under HPP conditions, they exhibited faster germination and greater susceptibility to inactivation.

Evidence has already shown that the occurrence of transcription and translation would precede CaDPA release during spore germination [56]. The release of DPA is accompanied by partial core hydration [57]. It remains unknown whether and how water enters the spore core during spore germination. Further study of the precise mechanism of water entry into spores should aim to further elucidate spore resistance properties and therefore innovate potential methods of spore inactivation.

## 5. Conclusions

In conclusion, this study has demonstrated that while *glpF* is not a critical determinant for initiating spore germination, it does influence the germination process. In addition, when we reduced the water activity and increased the osmotic pressure of the solution, we observed that the absence of *glpF* accelerated spore germination, consistent with the results observed in pure water. And GlpF overexpression affected the permeability of the spore coat. These findings strongly suggested that *glpF* should affect key processes in sporulation, which could lead to faster germination. And the lack of GlpF made the spores easier to kill with the PT treatment, which also provides inspiration for the innovation of ultra-high-pressure sterilization technology. In the future, the mechanism by which GlpF affects spore germination will be further investigated. 

## Figures and Tables

**Table 1:** Bacterial strains and plasmids used in this study.

Bacterial Strains	Genetype	Description	Reference
*B. subtilis* PY79	Wild type		Laboratory collection
BS01	*glpF::kan*	PY79 strain transformed with the DNA fragment containing *kan^R^* and the upstream and downstream homologous arms of *glpF*.	This study
BS02	*amyE::P_IPTG_-glpF spc*	PY79 strain transformed with the pBS1 plasmid.	This study
Plasmids	Genotype	Description	Reference
pDR111	*amyE::P_IPTG_ spc*		Laboratory collection
pBS1	*amyE::P_IPTG_-glpF spc*	pDR111 derivative plasmid encoding *glpF* from P_hyper-spank_	This study

## Data Availability

The original contributions presented in this study are included in the article. Further inquiries can be directed to the corresponding author.
